# Preoperative edema severity affects outcomes after Descemet membrane endothelial keratoplasty for Fuchs endothelial corneal dystrophy: a cohort study

**DOI:** 10.1186/s40662-025-00425-5

**Published:** 2025-03-01

**Authors:** Maximilian Friedrich, Hyeck-Soo Son, Jasper Lind, Maximilian Hammer, Lizaveta Chychko, Timur Mert Yildirim, Gerd Uwe Auffarth, Victor Aristide Augustin

**Affiliations:** Department of Ophthalmology, David J. Apple International Laboratory for Ocular Pathology and International Vision Correction Research Centre (IVCRC), University of Heidelberg, Im Neuenheimer Feld 400, 69120 Heidelberg, Germany

**Keywords:** Fuchs endothelial corneal dystrophy, Subclinical corneal edema, DMEK, Descemet membrane endothelial keratoplasty, Densitometry

## Abstract

**Background:**

In patients with Fuchs endothelial corneal dystrophy (FECD), the most beneficial stage to perform Descemet membrane endothelial keratoplasty (DMEK) remains uncertain. The goal of this study was to compare the surgical outcomes after DMEK in FECD patients with subclinical corneal edema and clinical corneal edema to test the hypothesis of whether performing surgery in subclinical corneal edema stages achieves better surgical outcomes.

**Methods:**

In this prospective, observational, single-institution cohort study, 106 pseudophakic eyes of 85 patients with FECD were divided into two groups depending on the presence of preoperative subclinical and clinical corneal edema. Subclinical corneal edema was diagnosed if more than one of the following criteria was present in Scheimpflug tomography: loss of regular isopachs, displacement of the thinnest point of the cornea, and focal posterior corneal surface depression. Clinical corneal edema was diagnosed with slit-lamp biomicroscopy. The primary outcome was the corrected distance visual acuity (CDVA) 4 months after DMEK. Secondary outcomes were central corneal thickness (CCT), thinnest corneal thickness (TCT), and total corneal density (TCD) in Scheimpflug tomography, as well as endothelial cell loss (ECL) and the re-bubbling rate. The differences between both groups were analyzed using clustered Wilcoxon rank-sum tests or a Chi-squared test.

**Results:**

Postoperative CDVA was significantly better in the group with subclinical edema (0.18 ± 0.12 logMAR) compared to the group with clinical edema (0.24 ± 0.19 logMAR; *P* = 0.026). Four months after DMEK, TCD was higher in the group with preoperative clinical edema [31.7 ± 8.3 gray scale units (GSU)] compared to the group with subclinical edema (27.8 ± 6.1 GSU; *P* = 0.005). The postoperative CCT, TCT, ECL, and re-bubbling rates did not differ significantly between both groups (all *P* > 0.05).

**Conclusions:**

DMEK for FECD yielded better visual acuity after 4 months when performed in the early stage of FECD compared to a later stage with clinical edema. This may be attributable to persistent corneal fibrosis after DMEK in eyes with preoperative clinically evident corneal edema, as suggested by higher postoperative corneal density in eyes with clinical edema. Consequently, the findings advocate for the consideration of earlier DMEK in FECD patients to achieve better surgical recovery.

**Supplementary Information:**

The online version contains supplementary material available at 10.1186/s40662-025-00425-5.

## Background

In patients with Fuchs endothelial corneal dystrophy (FECD) [1], the most common endothelial dystrophy [2], the visual function including contrast sensitivity and visual acuity becomes progressively compromised and straylight values tend to increase [3–6]. Descemet membrane endothelial keratoplasty (DMEK) is currently the gold standard in treating FECD [1, 7].

Using the modified Krachmer scale, the severity of FECD can be classified by slit-lamp biomicroscopy depending on the distribution pattern of corneal guttae and the presence of clinical corneal edema [8, 9]. In the most advanced Grade 6 of the modified Krachmer scale, corneal edema is biomicroscopically observable and strongly impedes visual function [10, 11]. However, Kopplin et al. reported that an increase in central corneal thickness (CCT) can be seen even in early stages of FECD without clinically observable edema, which indicates the presence of subclinical edema [12].

Thanks to modern Scheimpflug and optical coherence tomography, corneal edema can be detected in earlier stages of the disease, even when the edema is not clinically visible, by assessing specific tomographic criteria [13, 14]. Subclinical corneal edema is known to reduce visual acuity as well as contrast sensitivity and increase high-order aberrations regardless of the amount of guttae [5, 6, 15]. Therefore, DMEK is known to be beneficial for patients with subclinical corneal edema. Patel et al. found that with more parameters for subclinical corneal edema in Scheimpflug tomography, the need for surgical intervention rises [16]. However, the visual outcome of an early DMEK in the subclinical corneal edema stage of FECD compared to an intervention at a later stage with clinical edema has not been studied sufficiently.

The purpose of this study was to analyze whether FECD patients with subclinical corneal edema have a better postoperative outcome after DMEK than patients with clinical corneal edema.

## Methods

**Fig. 1:**
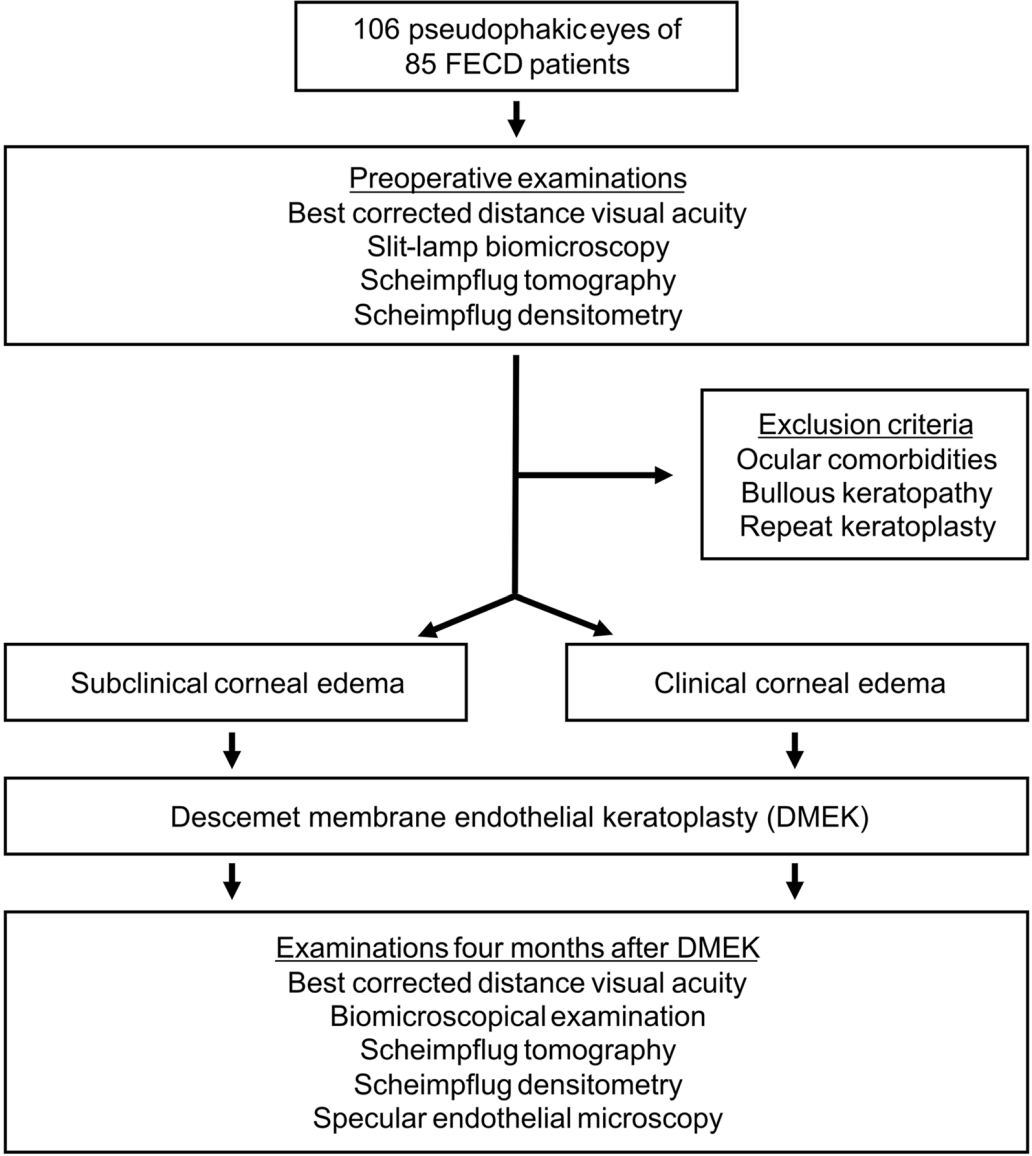
Study design

A total of 106 eyes of 85 patients with FECD were included in this prospective, observational, single-institution cohort study. The study design is shown in Fig. 1. All eyes were pseudophakic with monofocal intraocular lenses to avoid confounding effects of the patients’ lens or cataract. Eyes that previously underwent ocular surgery other than uncomplicated cataract surgery and eyes with a multifocal intraocular lens or other ocular comorbidities were excluded. Additionally, all eyes that underwent combined DMEK with cataract surgery (triple-DMEK) and those that received DMEK due to pseudophakic bullous keratopathy or failed endothelial keratoplasty were excluded.

This study was approved by the Institutional Review Board/Ethics Committee (ID: S-565/2023) at the Ruprecht-Karls University Heidelberg, Germany, and performed in accordance with the tenets of the Declaration of Helsinki. Informed consent was obtained from all participants.

### Preoperative measurements

All eyes underwent slit-lamp biomicroscopy and presented either with FECD Grade 5 on the modified Krachmer scale [8] with confluent guttae over 5 mm in diameter and no clinically evident corneal edema or an FECD Grade 6 with clinically visible corneal edema (Supplementary Fig. 1). The corrected distance visual acuity (CDVA) was preoperatively measured for each eye in the morning under photopic conditions (320 cd/m^2^) using an electronic 5-letter per-line chart at 5-m test distance.

All eyes were examined using Scheimpflug tomography (Pentacam AXL, Oculus Optikgeräte, Wetzlar, Germany) and analyzed via the *4 Maps Refractive* output as well as the *Corneal Densitometry* output. To detect subclinical corneal edema in eyes without clinical corneal edema, the following three criteria were analyzed by the observers (M.F. and V.A.A.) in each eye individually in accordance with the classification published by Sun et al. [13]:

1. Loss of regular parallel isopachs;
2. Displacement of the thinnest point of the cornea;
3. Focal posterior surface depression of the cornea.

**Fig. 2:**
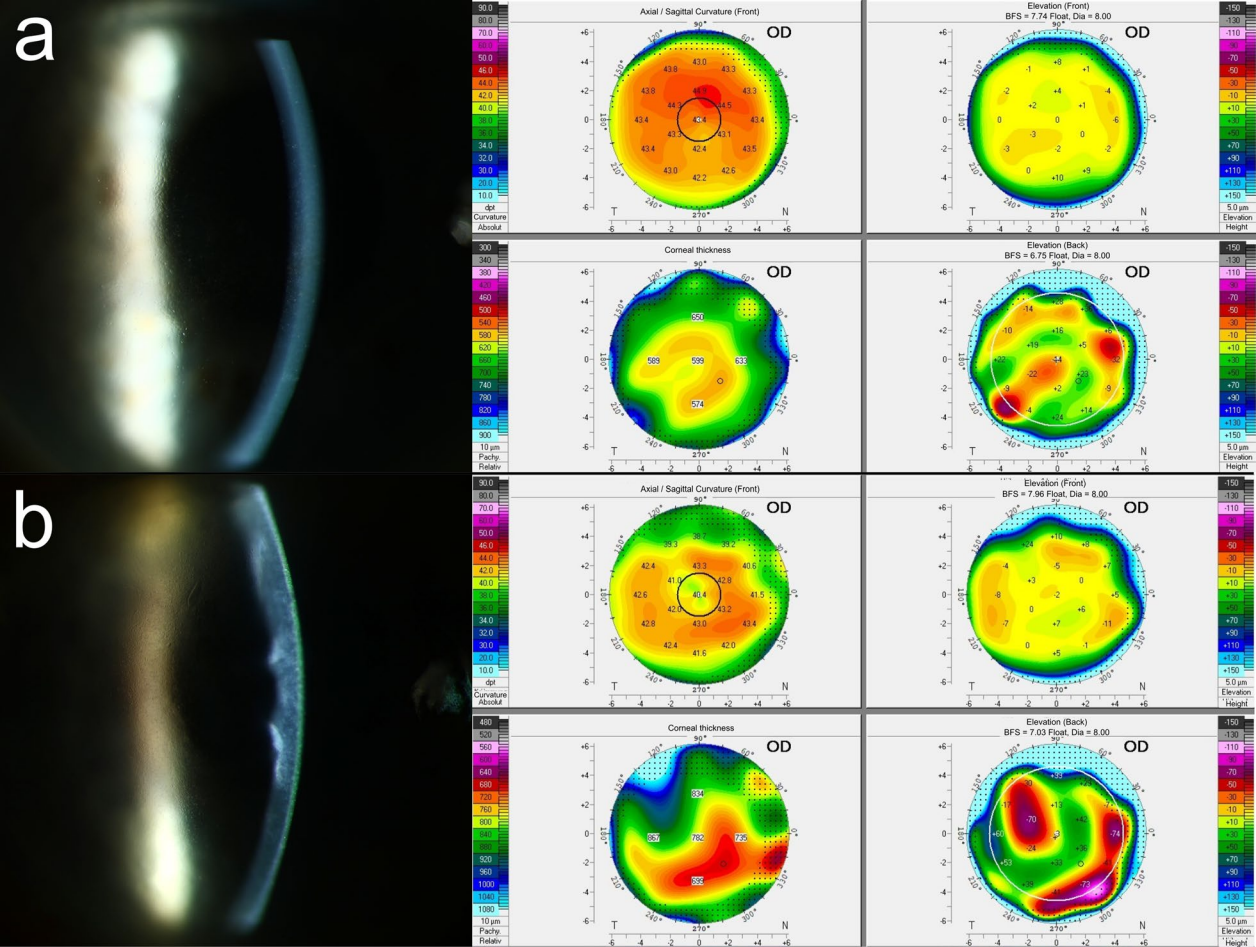
Examples for detection of subclinical corneal edema and clinical corneal edema. **a** The eye classified as having subclinical edema with all three criteria present in Scheimpflug tomography (focal posterior surface depression, loss of regular isopachs, and displacement of the thinnest point of the cornea). In slit-lamp biomicroscopy, corneal guttae are visible but no clinical corneal edema can be observed. **b** The eye classified as having clinical corneal edema showing diffuse corneal edema in slit-lamp biomicroscopy. In Scheimpflug tomography, higher pachymetry as well as higher irregularity can be observed

If two or three criteria were present, the eye was classified as ‘with subclinical corneal edema’ and was included in this study. If one or no criterion was present, the eye was excluded from this study as no clinical or subclinical edema was diagnosed. Figure 2 provides a visualization of slit-lamp biomicroscopy and corneal tomography of eyes with subclinical and clinical corneal edema.

Additionally, CCT, thinnest corneal thickness (TCT), and corneal volume were obtained from the *4 Maps Refractive* tomography output. From the *Corneal Densitometry* output, total corneal density (TCD) as well as the densitometry measurements divided by surface area and corneal layer were obtained. The densitometry values were analyzed in gray scale units (GSU), with minimum densitometry corresponding to 0 GSU and maximum densitometry to 100 GSU.

### Surgical procedure

A Nd:YAG laser iridotomy was performed at the 6 and 12 o’clock positions 1 day before surgery to minimize the risk of pupillary blockage after DMEK. All surgeries were performed by the same experienced surgeon (V.A.A.) under general anesthesia. The graft was prepared by the surgeon (V.A.A.) immediately prior to surgery using the previously described stripping technique [17, 18]. A 9 mm descemetorhexis was performed under air and the graft was injected using a Viscoject-Bio 2.2 cartridge (Medicel AG, Altenrhein, Switzerland) with an injector. The graft was unfolded by corneal tapping. To quantify the surgical difficulty, the time to unfold the graft in the anterior chamber was measured. After successful unfolding and central positioning of the corneal graft, 100% air tamponade was performed and left for one minute. Then, the anterior chamber was filled with a 20% sulfur hexafluoride (SF6) gas-air-mixture, covering 90% of the horizontal corneal diameter. All patients were postoperatively instructed to maintain a supine position to maximize the bubble graft coverage [19], and reduce complications such as graft detachment or increased intraocular pressure.

### Postoperative measurements

In the postoperative period, all incidents such as graft detachment or increased intraocular pressure were documented. If the graft was shown to be detached in more than 30% of the graft area in anterior segment optical coherence tomography (Anterion, Heidelberg Engineering, Heidelberg, Germany), a re-bubbling with 20% SF6 gas-air-mixture was performed under topical anesthesia in the operating room.

At the routine follow-up 4 months after DMEK, visual acuity was measured again as described above. Additionally, Scheimpflug tomography was performed again to measure the postoperative decrease in CCT, TCT, and corneal density. Endothelial cell density (ECD) in the central cornea was measured by a specular microscope (CEM-530, NIDEK, Gamagori, Aichi, Japan). The difference between the ECD of the graft before transplantation and the ECD 4 months after DMEK is the endothelial cell loss (ECL).

### Statistical analysis

Statistical analyses were performed with SPSS for Windows (version 29, IBM, Armonk, New York, USA) and R statistical software (version 4.2.2, R Foundation for Statistical Computing, Vienna, Austria) using the R package “clusrank” [20]. We performed clustered Wilcoxon rank-sum tests using the Datta-Satten method [21] for comparison of metric variables to account for the inclusion of both eyes of a patient in some cases. The primary outcome was the CDVA 4 months after surgery with a significance level of 0.05. Secondary outcomes were ECL, CCT, TCT, TCD, and re-bubbling rate. The difference in re-bubbling rate as well as descriptive patient characteristics were analyzed using the Chi-squared test. The sample size calculation to find a significant difference in CDVA with anticipated means of 0.1 ± 0.15 logarithm of the minimum angle of resolution (logMAR) in the group with subclinical corneal edema and 0.2 ± 0.15 logMAR in the group with clinical corneal edema (α = 0.05; β = 0.8; enrollment ratio 1:1) resulted in at least 39 eyes for each group.

## Results

The group with clinical corneal edema consisted of 59 eyes, while the group with subclinical corneal edema consisted of 47 eyes. In 40 of the 47 eyes (85.1%) with subclinical corneal edema, all three tomographical criteria were present. In the other seven eyes (14.9%), two out of the three criteria were present. The interobserver agreement regarding the classification was generally high (95.7%), and small discrepancies were resolved by a joint assessment of the authors. The characteristics of the study patients are displayed in Table 1 and did not differ significantly between both groups. The graft unfolding time was similar in both groups with a mean of 2.90 ± 2.15 min in the subclinical corneal edema group and 2.78 ± 1.87 min in the clinical corneal edema group (*P* = 0.789).

**Table 1:** Characteristics of all study eyes, classified by the presence of subclinical or clinical corneal edema

Parameter		Subclinical corneal edema	Clinical corneal edema	*P* value
Eyes (n)	Total	47	59	–
Gender (n)	Women	31	28	0.06
	Men	16	31	
Age (years, mean ± SD)		71.6 ± 9.0	72.4 ± 9.0	0.24
Eyes (n)	Right	30	34	0.52
	Left	17	25	
Number of re-bubblings (n)	None	40	49	0.92
	1	6	8	
	2	1	2	

### Visual acuity

**Fig. 3:**
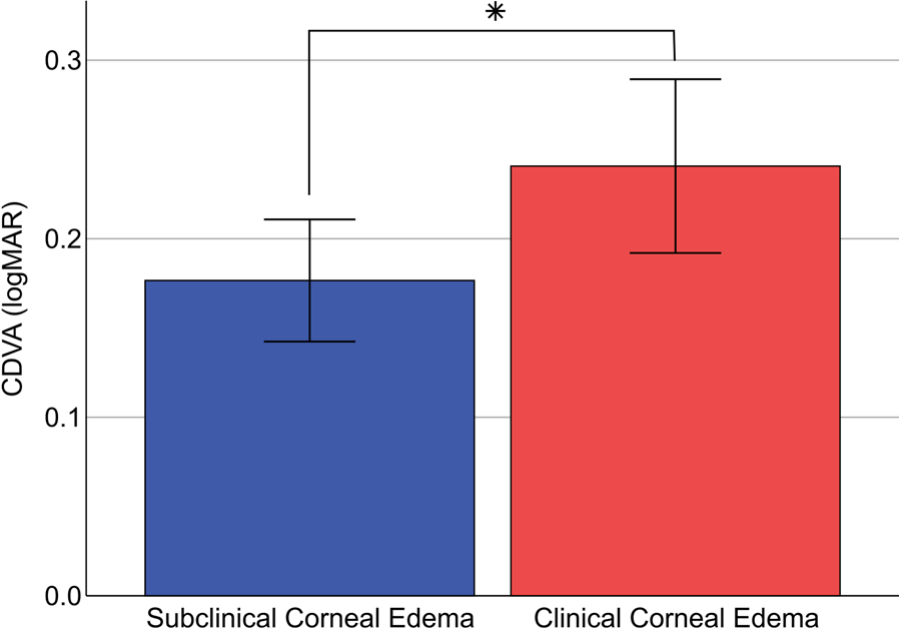
Corrected distance visual acuity 4 months after Descemet membrane endothelial keratoplasty (DMEK) depending on edema severity. Error bars = 95% confidence interval. CDVA, corrected distance visual acuity; logMAR, logarithm of the minimum angle of resolution. *P < 0.05

The CDVA 4 months after DMEK was significantly better in eyes with preoperative subclinical corneal edema (0.18 ± 0.12 logMAR) compared to eyes with preoperative clinical corneal edema (0.24 ± 0.19 logMAR; *P* = 0.026) as shown in Fig. 3. The mean time of measurement after DMEK was 115.9 days in the group with subclinical corneal edema and 132.8 days in the group with clinical corneal edema. 93.6% of all cases with subclinical edema presented with a postoperative CDVA equal or better than 0.3 logMAR, compared to 79.7% of all cases with clinical edema. As expected, the preoperative CDVA was significantly worse in the group with clinical edema than in the group with subclinical edema (*P* < 0.001; see Table 2).

**Table 2:** Descriptive statistics of the examined parameters (mean ± SD) before and 4 months after Descemet membrane endothelial keratoplasty (DMEK) in the subclinical and clinical corneal edema groups

Parameter	Subclinical corneal edema		Clinical corneal edema	
	Before DMEK	After DMEK	Before DMEK	After DMEK
Corrected distance visual acuity (logMAR)	0.35 ± 0.12	0.18 ± 0.12	0.54 ± 0.27	0.24 ± 0.19
Endothelial cell density* (cells/mm^2^)	2562.9 ± 199.9	1772.1 ± 505.3	2558.7 ± 195.1	1557.0 ± 524.8
Central corneal thickness (µm)	606.8 ± 50.0	516.9 ± 36.5	667.2 ± 89.7	514.7 ± 36.7
Thinnest corneal thickness (µm)	583.6 ± 46.9	508.8 ± 38.7	613.5 ± 50.2	506.0 ± 37.0
Total corneal density (GSU)	26.4 ± 5.9	27.8 ± 6.1	33.1 ± 9.8	31.7 ± 8.3

*logMAR* = logarithm of the minimum angle of resolution; *GSU* = gray scale units

^*^Before DMEK, the endothelial cell density of the donor graft was measured

### Pachymetry

**Fig. 4:**
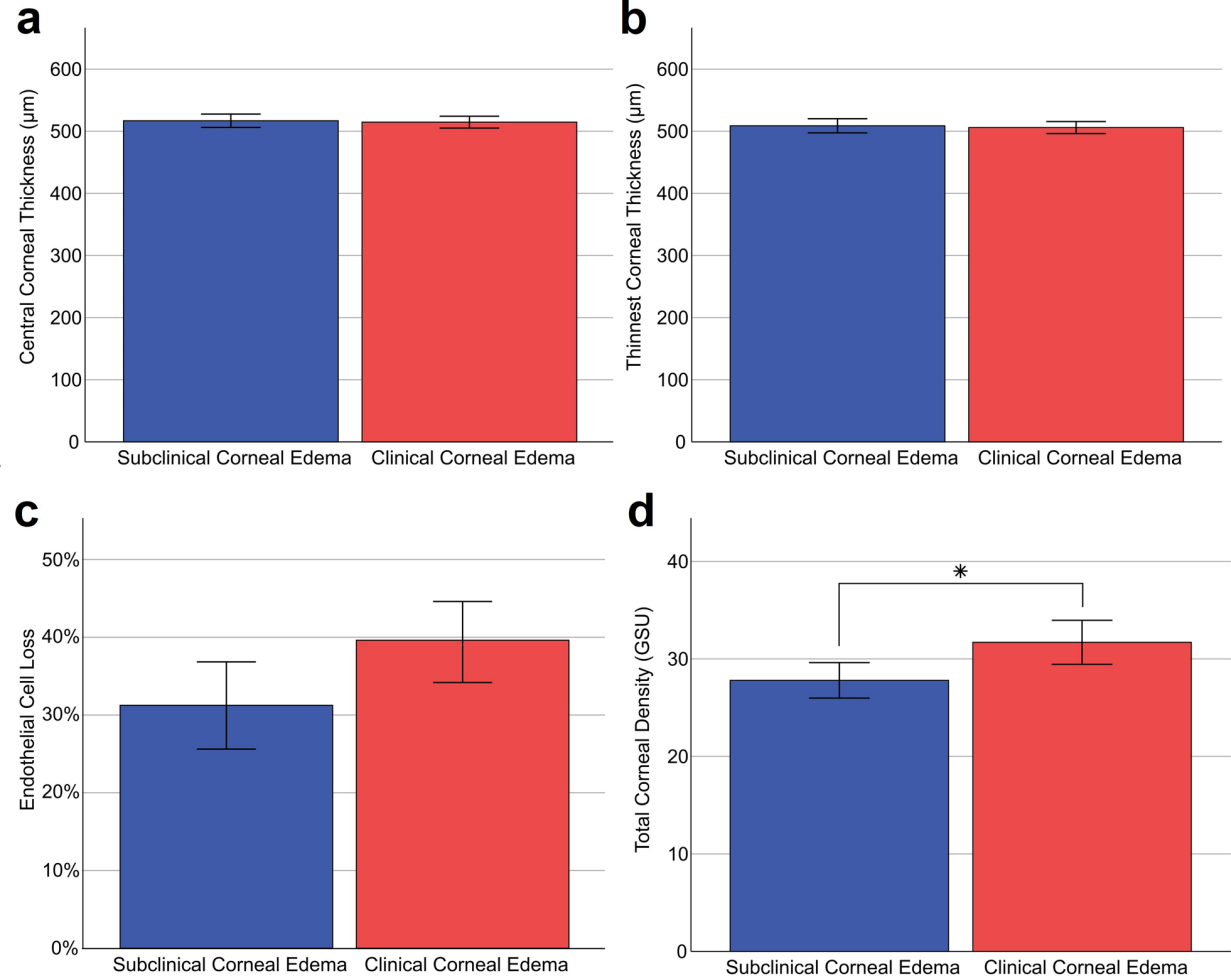
Secondary outcome parameters 4 months after Descemet membrane endothelial keratoplasty (DMEK) depending on edema severity. **a** Central corneal thickness measured by Scheimpflug tomography. **b** Thinnest corneal thickness measured by Scheimpflug tomography. **c** Endothelial cell loss calculated by dividing the endothelial cell density measured with a specular microscope 4 months after DMEK by the preoperative endothelial cell density of the graft. **d** Total corneal density measured by Scheimpflug photography. Blue colored bars represent FECD eyes with preoperative subclinical corneal edema. Red colored bars represent FECD eyes with preoperative clinical corneal edema. Error bars = 95% confidence interval. GSU, gray scale units. *P < 0.05

The CCT 4 months after DMEK did not differ significantly (*P* = 0.404) between groups with a mean of 516.9 ± 5.3 µm and 514.7 ± 4.8 µm in the group with subclinical and clinical edema, respectively (Fig. 4a). Similarly, the TCT 4 months after DMEK did not differ significantly (*P* = 0.410) with a mean of 508.8 µm and 506.0 µm, respectively (Fig. 4b). However, the preoperative CCT (*P* < 0.001) and TCT (*P* = 0.002) were significantly higher in the group with clinical corneal edema compared to the group with subclinical corneal edema. The preoperative corneal volume was significantly higher in the group with clinical edema (63.9 ± 6.9 mm^3^) than in the group with subclinical edema (60.5 ± 4.2 mm^3^; *P* = 0.014). Postoperatively, the corneal volume was comparable in both groups (59.9 ± 6.1 mm^3^ and 59.1 ± 4.3 mm^3^).

### Densitometry

**Fig. 5:**
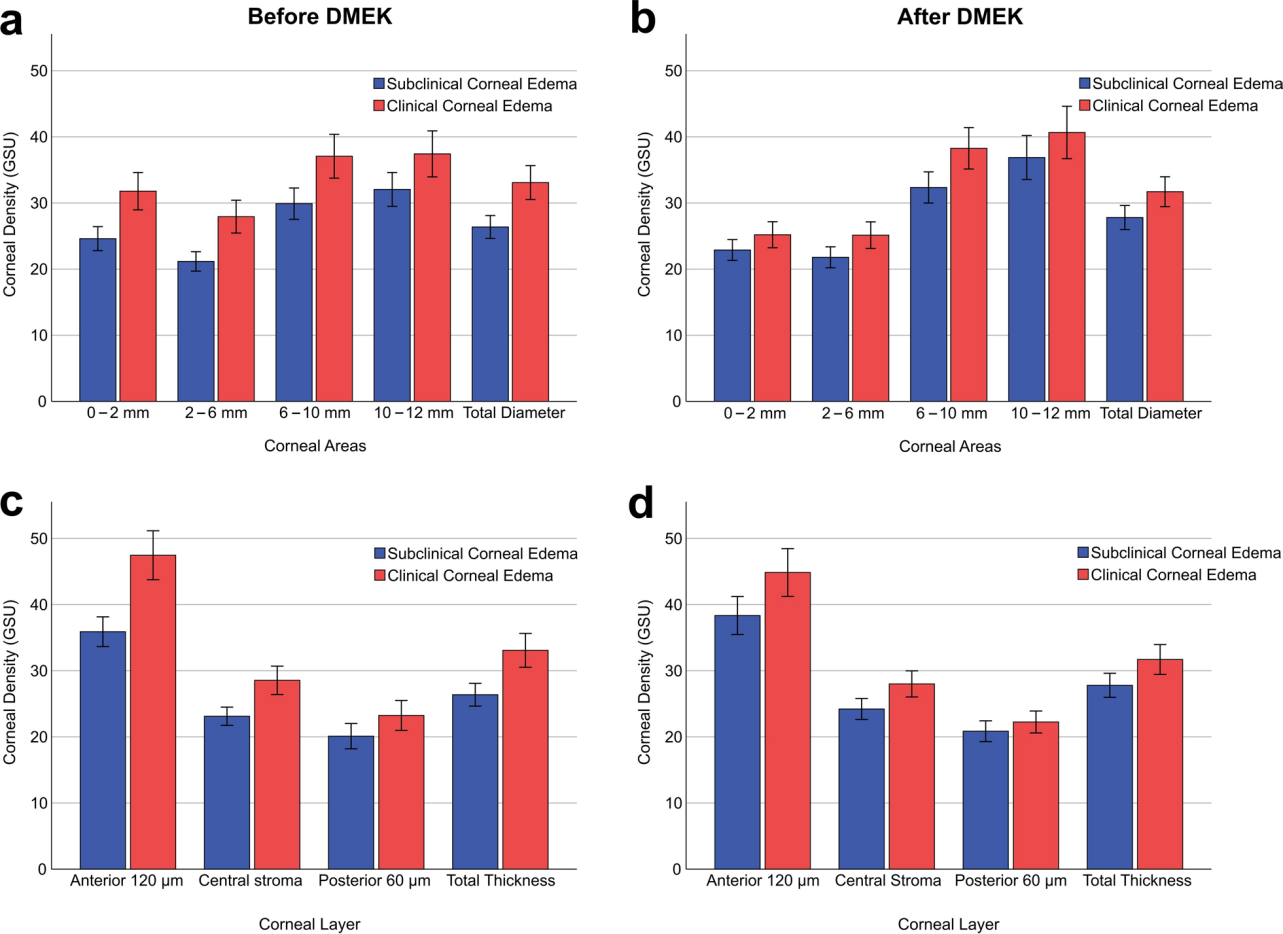
Corneal densitometry preoperatively and 4 months after Descemet membrane endothelial keratoplasty (DMEK) depending on edema severity. **a** Preoperative corneal density depending on the area of measurement divided into five annuli ranging from the most central surface (0–2 mm) to the most peripheral annulus (10–12 mm). **b** Corneal density depending on the area of measurement 4 months after DMEK divided into five annuli ranging from the most central surface (0–2 mm) to the most peripheral annulus (10–12 mm). **c** Preoperative corneal density depending on the layer of measurement divided into four layers: the anterior 120 µm of the cornea, the posterior 60 µm of the cornea and the central stroma between both layers with variable thickness depending on the total corneal thickness. **d** Corneal density depending on the layer of measurement 4 months after DMEK divided into three layers: the anterior 120 µm of the cornea, the posterior 60 µm of the cornea and the central stroma between both layers with variable thickness depending on the total corneal thickness. Blue colored bars represent Fuchs endothelial corneal dystrophy (FECD) eyes with preoperative subclinical corneal edema. Red colored cards reperesent FECD eyes with preoperative clinical corneal edema. Error bars = 95% confidence interval

The TCD 4 months after DMEK was significantly higher in the group with clinical corneal edema (31.7 ± 8.3 GSU) than in the group with subclinical corneal edema (*P* = 0.005; 27.8 ± 6.1 GSU; Fig. 4d). Preoperatively, the total corneal density also differed significantly (*P* < 0.001) with a mean of 26.4 ± 5.9 GSU and 33.1 ± 9.8 GSU in the group with subclinical and clinical edema, respectively. The corneal density subdivided by area and corneal layer is shown in Fig. 5. Preoperatively, corneal density in eyes with subclinical edema was lower in all areas (Fig. 5a) and all layers (Fig. 5c) when compared to eyes with clinical edema. A similar trend was observed 4 months after DMEK (see Fig. 5b, d).

### Endothelial cell loss

The mean ECL 4 months after DMEK was 30.9 ± 18.3% in the group with subclinical edema and 39.2% ± 20.0% in the group with clinical edema (Fig. 4c). The difference in ECL was not statistically significant (*P* > 0.05). The mean pre- and postoperative endothelial cell densities are shown in Table 2.

### Complications

In the group with subclinical edema, 7 out of 47 eyes (14.9%) had at least one re-bubbling with one case having two subsequent re-bubblings due to persistent graft detachment. In the group with clinical edema, 10 out of 59 eyes (16.9%) had at least one re-bubbling with two cases having two subsequent re-bubblings. However, the re-bubbling rates between the two groups did not differ significantly (*P* = 0.774). In all cases, no significant rise in intraocular pressure during the postoperative period was observed.

## Discussion

This study demonstrates that the visual acuity 4 months after DMEK is significantly better in FECD eyes that had a preoperative subclinical corneal edema compared to eyes with a preoperative clinical corneal edema. The findings may be important when counseling patients on when to undergo endothelial keratoplasty to reach optimal visual outcomes after DMEK in the early postoperative period.

The inferior postoperative CDVA in the group with clinical corneal edema could be explained by corneal fibrosis. The current study found that postoperative TCD as well as most subgroup analyses of the surface areas and the corneal layers were denser in the group with preoperative clinical edema compared to the group with subclinical edema after DMEK. These indicate that eyes with preoperative clinical edema have an opaquer cornea 4 months after DMEK, which may explain their difference in CDVA. Compared to the normative study from Ní Dhubhghaill et al. which found a mean total corneal density of 19.74 ± 3.89 GSU in healthy patients [22], both groups showed a higher density, yet the clinical edema group presented with significantly higher postoperative values (31.7 ± 8.3 GSU). This increase in density, which is backscatter measured by the Scheimpflug camera, indicates corneal haze [23]. In vivo laser confocal microscopy showed a subtle subepithelial and interface haze after DMEK in eyes with bullous keratopathy [24], which highlights the possibility of corneal fibrosis after DMEK. Therefore, a possible explanation for the increase in corneal density is the development of corneal fibrosis resulting from a longer prevailing and intensified corneal edema in patients with more advanced stages of FECD. Future studies with confocal microscopy are necessary to confirm whether corneal fibrosis indeed occurs in cases of higher preoperative edema severity.

Our results show that surgical intervention at earlier stages of FECD may lead to better visual outcomes, and thus DMEK may preferably be performed at the subclinical corneal edema stage. Favoring earlier DMEK has also been proposed by other clinical studies. Schrittenlocher et al. stated that a preoperative visual acuity below 0.7 logMAR results in a delayed recovery and reduced final visual acuity results after 12 months [25]. Our study showed that even with a mean preoperative visual acuity of 0.54 logMAR in the clinical corneal edema group, early visual recovery is not as good as in eyes with a better preoperative visual acuity. Yet, additional studies with a longer follow-up are needed to differentiate between a faster visual recovery vs. superior long-term visual outcome in the subclinical edema group after DMEK.

A retrospective study showed that visual rehabilitation is even better in FECD eyes without any corneal edema compared to eyes with subclinical edema, underlining the potential benefit of an early intervention [26]. Since subclinical edema itself reduces visual acuity as well as contrast sensitivity [5], patients may benefit from an earlier DMEK in the subclinical edema stage when considering multiple components of visual function. Another study found that DMEK improves visual function even in the early postoperative period leading to an increase in visual acuity and contrast sensitivity although not entirely reaching the visual function of healthy controls [27]. Nevertheless, an observational study by Bayyoud et al. showed that DMEK can significantly improve the logarithmic contrast sensitivity measured with a Pelli-Robson chart even in mildly impaired eyes [28]. Additionally, the amount of straylight can be improved with endothelial keratoplasty [29]. Future studies on the stage-dependent visual outcome after DMEK should address the influence of preoperative edema severity on other parameters of the visual function such as contrast sensitivity and straylight to gather more insight on the benefits of an earlier surgical intervention.

Preoperative pachymetry was found to be significantly higher in the clinical edema group. However, corneal pachymetry improved in all eyes after DMEK and did not significantly differ between both groups postoperatively. A similar trend was observed by another study comparing pachymetry values after DMEK of FECD eyes with clinical, subclinical, and no corneal edema [26]. The improvement in pachymetry due to DMEK even in the subclinical edema stage has also been previously described by Sun et al. when introducing the classification for subclinical edema [13]. These results show that the classification for subclinical corneal edema [13] successfully identifies eyes without clinical edema that may benefit from DMEK in terms of pachymetry and, as shown in the results above, in terms of visual acuity.

The ECL as well as the complication rate did not significantly differ between both groups in this study. In comparison to recent reviews describing the postoperative complication rates after uncomplicated DMEK [29–31], the results of this study are comparable. When consulting patients in the early stages of FECD, corneal surgeons should carefully assess the risk–benefit balance between improved visual outcomes at early-stage intervention and a 14.9% re-bubbling rate after DMEK to support informed decision-making.

Our study is not without its limitations. As the study was only powered for the primary outcome CDVA, statistically significant results in secondary outcomes have only exploratory value and need to be confirmed in a different study population. A loss in CDVA may also occur due to many other ocular diseases such as cataract or glaucoma. To limit the influence of lenticular causes and other comorbidities on the visual acuity, we only included pseudophakic eyes and excluded eyes with any other ocular comorbidities. The analyzed follow-up was somewhat short, indicating a faster recovery in eyes with subclinical edema. No clinical corneal edema was observed 4 months after surgery in all eyes with preoperative clinical corneal edema. However, the study will be continued to confirm our findings in later follow-up examinations. Additionally, all surgeries were performed by the same experienced surgeon (V.A.A.), which could be a confounding variable leading to different results for other surgeons. Furthermore, while the tomographic detection of subclinical corneal edema may be partly subjective, the masked interobserver agreement was high with an identical classification in 95.7% of the eyes. Finally, we included both eyes of some patients in this study, which could confound statistical analysis due to the possible interdependence of fellow eyes. To address the clustered nature of the data, we used clustered Wilcoxon rank-sum test as our tool of choice for analyzing differences [21].

## Conclusions

DMEK yielded better visual acuity when performed in eyes with subclinical edema compared to eyes with clinical edema. The difference in CDVA may be attributable to the presence of corneal fibrosis in eyes with clinically evident corneal edema, as suggested by higher postoperative corneal density in eyes with preoperative clinical edema. However, additional studies with a longer follow-up are needed to confirm these findings. The better visual outcome in eyes with preoperative subclinical edema may be an advocate for endothelial keratoplasty in the earlier stages of FECD. Nevertheless, other patient-related variables should also be considered such as patient-reported symptoms when deciding whether a surgical intervention is advantageous for a patient. Future studies should investigate the benefits of early endothelial keratoplasty on other visual parameters such as contrast sensitivity and straylight in FECD eyes depending on corneal edema severity to promote a standardized and evidence-based treatment for FECD patients.

## Supplementary Information

Supplementary material 1: Figure 1. Comparison of Krachmer grade 5 and Krachmer grade 6 in eyes with Fuchs endothelial corneal dystrophy. **a** Krachmer grade 5 in retroillumination (confluent corneal guttae over 5 mm or more without corneal edema). **b** Krachmer grade 6 in retroillumination (clinical corneal edema).

## Data Availability

The datasets generated and/or analyzed during this study are not publicly available but may be obtained from the corresponding author upon reasonable request.

## Abbreviations

CDVA: Corrected distance visual acuity
CCT: Central corneal thickness
DMEK: Descemet membrane endothelial keratoplasty
ECD: Endothelial cell density
ECL: Endothelial cell loss
FECD: Fuchs endothelial corneal dystrophy
GSU: Gray scale units
logMAR: Logarithm of the minimum angle of resolution
SF6: Sulfur hexafluoride
TCD: Total corneal density
TCT: Thinnest corneal thickness
