# Supplementary figures and images for: Preoperative edema severity affects outcomes after Descemet membrane endothelial keratoplasty for Fuchs endothelial corneal dystrophy: a cohort study
Source: Eye Vis (Lond). 2025 Mar 1;12:9. doi: 10.1186/s40662-025-00425-5 (PMC11871603; doi:10.1186/s40662-025-00425-5)

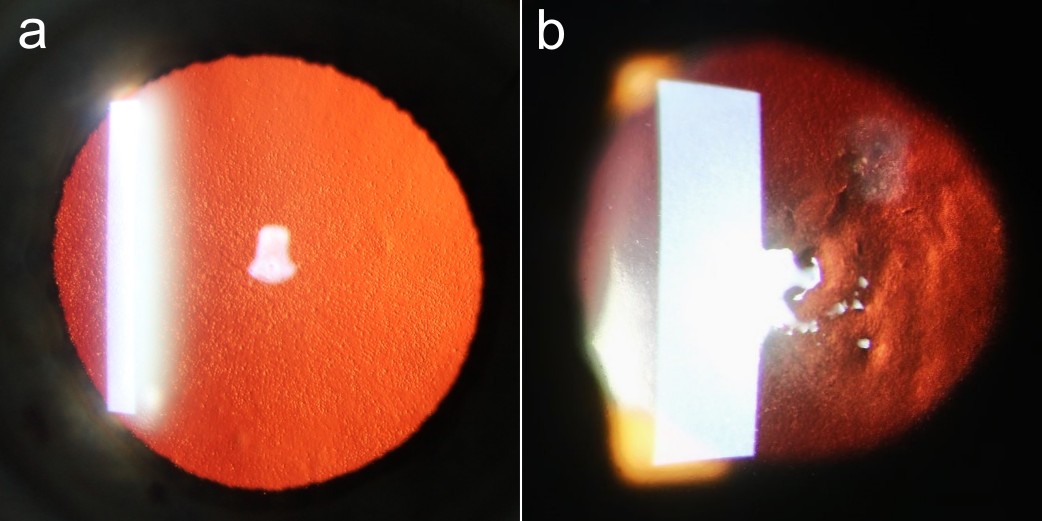

Supplement: Supplementary file 1 — Supplementary material 1: Figure 1. Comparison of Krachmer grade 5 and Krachmer grade 6 in eyes with Fuchs endothelial corneal dystrophy. a Krachmer grade 5 in retroillumination (confluent corneal guttae over 5 mm or more without corneal edema). b Krachmer grade 6 in retroillumination (clinical corneal edema). [file 40662_2025_425_MOESM1_ESM.jpg]
